# Controlling J-Aggregates Formation and Chirality Induction through Demetallation of a Zinc(II) Water Soluble Porphyrin

**DOI:** 10.3390/ijms21114001

**Published:** 2020-06-03

**Authors:** Ilaria Giuseppina Occhiuto, Maria Angela Castriciano, Mariachiara Trapani, Roberto Zagami, Andrea Romeo, Robert F. Pasternack, Luigi Monsù Scolaro

**Affiliations:** 1 Dipartimento di Scienze Chimiche, Biologiche, Farmaceutiche ed Ambientali, University of Messina and C.I.R.C.M.S.B V.le F. Stagno D’Alcontres, 31-98166 Messina, Italy; iocchiuto@unime.it; 2 CNR-ISMN Istituto per lo Studio dei Materiali Nanostrutturati c/o Dipartimento di Scienze Chimiche, Biologiche, Farmaceutiche ed Ambientali, University of Messina, V.le F. Stagno D’Alcontres, 31-98166 Messina, Italy; mariachiara.trapani@cnr.it (M.T.); roberto.zagami@ismn.cnr.it (R.Z.); 3 Department of Chemistry, Swarthmore College, 500 College Avenue, Swarthmore, PA 19081, USA; rpaster1@swarthmore.edu

**Keywords:** J-aggregates, symmetry breaking, demetallation kinetics, aggregation kinetics, chiral supramolecular assemblies

## Abstract

Under acidic conditions and at high ionic strength, the zinc cation is removed from its metal complex with 5,10,15,20-tetrakis(4-sulfonatophenyl)porphyrin (TPPS_4_) thus leading to the diacid free porphyrin, that subsequently self-organize into J-aggregates. The kinetics of the demetallation step and the successive supramolecular assembly formation have been investigated as a function of pH and ionic strength (controlled by adding ZnSO_4_). The demetallation kinetics obey to a rate law that is first order in [ZnTPPS_4_] and second order in [H^+^], according to literature, with *k*_2_ = 5.5 ± 0.4 M^−2^ s^−1^ at 298 K (IS = 0.6 M, ZnSO_4_). The aggregation process has been modeled according to an autocatalytic growth, where after the formation of a starting seed containing *m* porphyrin units, the rate evolves as a power of time. A complete analysis of the extinction time traces at various wavelengths allows extraction of the relevant kinetic parameters, showing that a trimer or tetramer should be involved in the rate-determining step of the aggregation. The extinction spectra of the J-aggregates evidence quite broad bands, suggesting an electronic coupling mechanism different to the usual Frenkel exciton coupling. Resonance light scattering intensity in the aggregated samples increases with increasing both [H^+^] and [ZnSO_4_]. Symmetry breaking occurs in these samples and the J-aggregates show circular dichroism spectra with unusual bands. The asymmetry g-factor decreases in its absolute value with increasing the catalytic rate *k_c_*, nulling and eventually switching the Cotton effect from negative to positive. Some inferences on the role exerted by zinc cations on the kinetics and structural features of these nanostructures have been discussed.

## 1. Introduction

J-aggregates of dyes originate from an edge-to-edge arrangement of chromophores in the self-assembled nanostructure. Their peculiar physical-chemical properties have attracted the attention of many research groups in the last decades [1,2]. Among the building blocks able to organize into these supramolecular architectures, water soluble sulfonato-porphyrins are well established. In particular, 5,10,15,20-tetrakis(4-sulfonatophenyl)porphyrin (TPPS_4_) has been largely investigated, since, depending on the experimental conditions, it is able to form a variety of structures spanning from the nano up to micro scale [3,4,5,6,7,8,9,10]. The expression of chirality in chromophoric, and more specifically in porphyrin supramolecular architectures, is a widely explored topic [11,12,13,14,15,16,17,18]. Usually, chiroptical properties could arise from the presence of stereogenic centers in the basic building blocks [19,20,21,22,23,24,25], or induced by a chiral scaffold or chemical template, such as simple chiral compounds, polymers and biomolecules [26,27,28,29,30,31,32,33,34,35]. Many examples of J-aggregated TPPS_4_ exhibiting optical activity under the presence of chemical [36,37,38,39,40,41,42,43,44,45,46,47] or physical chiral bias [48,49,50,51,52,53,54,55] have been reported so far. The occurrence of spontaneous symmetry breaking during the growth process has been related to kinetics and in particular to mixing protocols [56]. Factors such as aging time, temperature, gradients of concentration and so on, have been pointed out as responsible for different kinetic pathways during the aggregation of colloidal systems leading to very different mesoscopic structures [53,57,58,59]. This evidence is related to the tendency to form dimers, small oligomers or larger species, whose size depends widely upon experimental conditions and directly controls the nucleation stage or early stage of the self-assembly formation. Even if the dependence on every single parameter could be envisaged, a combination of all these factors makes the starting state a quite unpredictable scenario. On these bases, in order to gain a precise control in the self-assembling process, it is very important to deal with stock solution of porphyrin in a monomeric state. In our previous investigations on the role of adventitious traces of zinc(II) cation in the formation of TPPS_4_ J-aggregates, we showed that the formation of relatively small quantities of the ZnTPPS_4_ metal derivative slows down the kinetics of self-assembling and exerts a considerable impact on the onset of chirality in the eventual aggregates [60]. Actually, the easy extrusion of Zn(II) from the porphyrin core is due to its lability under acidic conditions; [61] leading, in solution, to the protonated monomeric porphyrin that is able to self-aggregate. Furthermore, due to the propensity to penta-coordination of this metal ion, the presence of a fifth ligand bound to the metal center hinders the formation of aggregated species, at least at rather low ZnTPPS_4_ concentrations. This strategy has proved to be successful in controlling the chirality of supramolecular assembly of this porphyrin driven by preorganization on a polypeptide template [30,62].

**Scheme 1:**
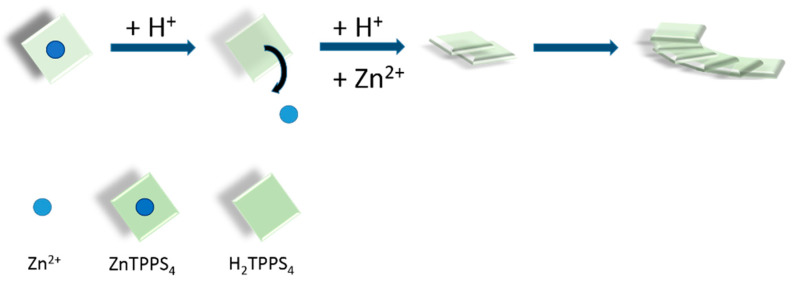
Pictorial sketch of ZnTPPS_4_ demetallation and subsequent aggregation of the diacid H_2_TPPS_4_.

Here we report a detailed kinetic investigation on the J-aggregates formation following demetallation of ZnTPPS_4_, as a function of pH and ionic strength (Scheme 1). We anticipate that the proper choice of the experimental conditions allows a fine control of the amount of free protonated porphyrins released over time in solution, thus affording a way to modulate the final chiroptical properties of the aggregates.

## 2. Results and Discussion

### 2.1. Kinetic Analysis

**Figure 1:**
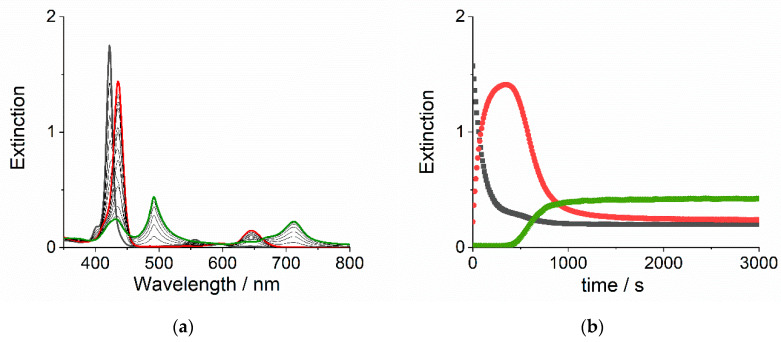
(**a**) UV/Vis extinction spectral changes for the demetallation of ZnTPPS_4_ (black) into H_2_TPPS_4_ (red) and the consecutive formation of J-aggregates (green), after the addition of sulfuric acid (experimental conditions: [ZnTPPS_4_] = 3 μM; [H_2_SO_4_] = 0.05 M, IS = 0.6 M by ZnSO_4_, T = 298 K, total acquisition time 3000 s). (**b**) Extinction time traces at 422, 434 and 490 nm for the same kinetic run.

As previously reported, in order to study aggregation kinetics of the parent TPPS_4_ we proposed two different approach: (i) porphyrin first, PF, where the aggregation is triggered on adding an acidic solution to prediluted TPPS_4_; or (ii) porphyrin last, PL, adding a small volume of stock solution of porphyrin to the acid at the final required concentration. On adopting these different mixing protocols to start aggregation, as a consequence of different nucleation pathways, the kinetic traces are deeply affected in their rates and in their general profiles, being sigmoidal (PF) or stretched exponentially (PL) [56]. In the present investigated case, the use of ZnTPPS_4_ as precursor of the diacid porphyrin is expected to afford a monomeric initial state, thus overcoming the issue of the mixing protocol. Indeed, the profiles and rates we obtained are independent of the mixing order and sigmoidal traces are observed in all the experiments. As the rate of demetallation is strongly dependent on the proton concentration, low pH required to initiate the aggregation of TPPS_4_ will cause an almost instantaneous release of the zinc cation. In order to gain better control of the formation of the monomeric diacid TPPS_4_ and to efficiently foster aggregation, we performed our experimental investigation at pH above 1 in the presence of added ZnSO_4_, to increase the ionic strength. The choice of this salt was dictated by: (i) the necessity of avoiding the introduction of ions different from those already present in solution, (ii) the already outlined lability of zinc(II) and (iii) considering that this salt has already been used in previous investigation on assembling and disassembling of J-aggregates [63,64]. When an aqueous solution of sulfuric acid is added to a diluted ZnTPPS_4_ solution in the presence of ZnSO_4_, a typical time evolution of the UV/Vis extinction spectra is displayed in Figure 1a. The B-band of ZnTPPS_4_ (3 μM) at 422 nm gradually decreases, matching the parallel increase of the B-band of the H_2_TPPS_4_ at 434 nm. This spectral feature reaches its maximum intensity and subsequently decreases with the matching increase of a new broad band at 490 nm that is assigned to the J-aggregate. The unusual aspect of this band, much broader with respect to that observed for J-aggregates obtained in the presence of simple inorganic acids and mono-cations (i.e., Na^+^ or K^+^), is reminiscent of that obtained in the presence of polycationic species, such as spermine [65,66,67,68]. The complete time evolution of the extinction at the selected wavelengths corresponding to the different species, ZnTPPS_4_ (λ = 422 nm), H_2_TPPS_4_ (λ = 434 nm) and J-aggregate (λ = 490 nm), is shown in Figure 1b.

**Figure 2:**
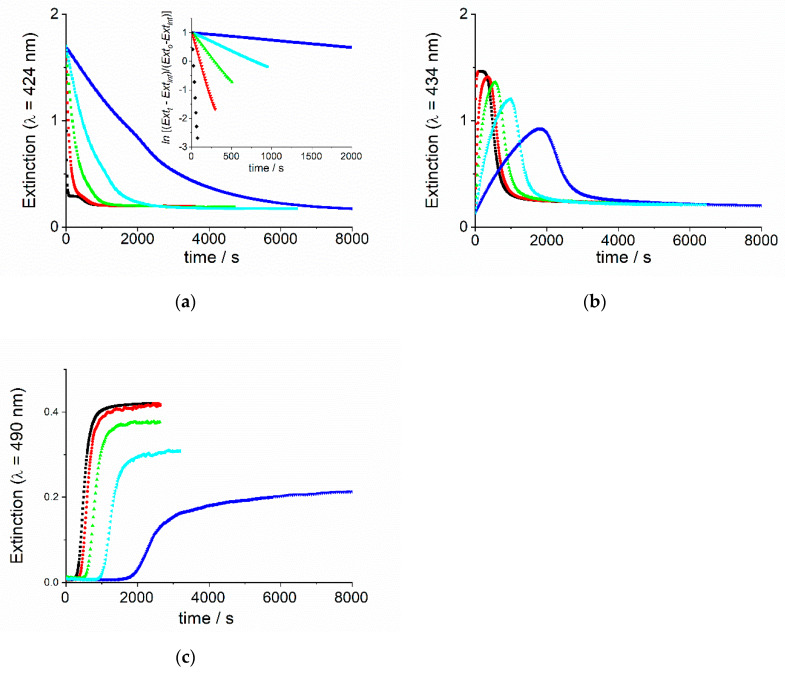
Extinction time traces for kinetic experiments on demetallation of ZnTPPS_4_ at constant ionic strength and different acid concentrations: (**a**) at 422 nm (in the inset the semilogarithmic plots according to a first order rate law are reported); (**b**) at 434 nm and (**c**) at 490 nm. (Experimental conditions: [ZnTPPS_4_] = 3 μM; [H_2_SO_4_] = 0.01 (blue), 0.02 (cyan), 0.03 (green), 0.05 (red) and 0.1 M (black), IS = 0.6 M by ZnSO_4_, T = 298 K).

**Figure 3:**
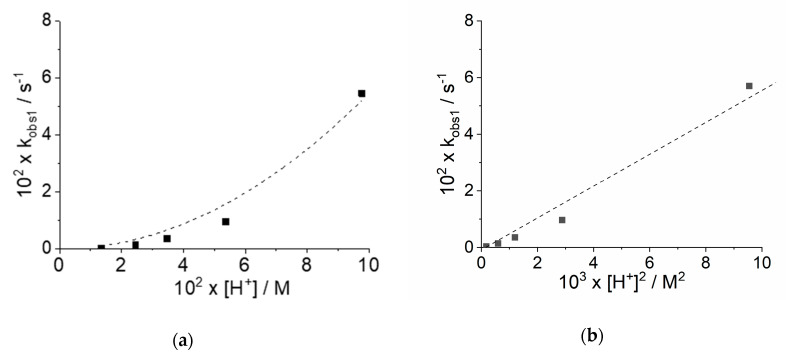
Plot of the pseudo-first order rate *k_obs_*_1_ (s^−1^) for the demetallation of ZnTPPS_4_ at constant ionic strength as function of (**a**) [H^+^] (dashed line is the best fit to the function *k_obs1_* = *k*_2_[H^+^]^2^) and (**b**) [H^+^]^2^ (dashed line is the linear fit of the experimental data).

Therefore, the kinetic analysis of the system under investigation has been performed at 298 K using the extinction time traces of these three bands, by changing the pH at fixed added salt concentration or at constant pH varying the added salt concentration. All the kinetic rate constants and related parameters are collected in Table 1. At [ZnSO_4_] = 0.6 M, an increase of proton concentration causes an acceleration of the ZnTPPS_4_ demetallation (Figure 2a). Since we adopted pseudo-first order conditions ([ZnTPPS_4_] << [H^+^]), the pseudo-first order observed rate constant (*k_obs_*_1_, s^−1^) can be obtained either through an exponential fit (Equation (1a)) of the extinction data at 422 nm vs. time or through the slope of a linear fit of a semi logarithm plot (Equation (1b)), inset Figure 2a, Table 1). According to literature, the demetallation process of ZnTPPS_4_ obeys to the rate law: rate = *k*_2_ [ZnTPPS_4_][H^+^]^2^ [69]. The values of *k_obs_*_1_ show a second order dependence on [H^+^] (Figure 3a), also confirmed by the linear behavior of these data as a function of [H^+^]^2^ (Figure 3b). A best fitting procedure to both models leads to a second order rate constant for the demetallation process of *k*_2_ = 5.5 ± 0.4 M^−2^ s^−1^, in good agreement with the literature value (*k*_2_ = 10.9 ± 0.6 M^−2^ s^−1^ at T = 303 K, IS = 0.1 M (NaClO_4_)). Under our experimental conditions, we failed to observe the expected dependence of *k_obs_*_1_ for the demetallation on the ionic strength, in other words, [ZnSO_4_] (Table 1). Indeed, earlier kinetic studies on this mechanism reported a dependence on the ionic strength and also on the nature of the counter-anions, especially for cationic porphyrins (where a second order dependence on anion concentration has been observed) [61,69]. Here, the invariance of the observed rates could be explained by a specific accelerating effect of the sulfate anion that is balancing the expected decreasing effect of increasing the ionic strength in a reaction between species of opposite charges. Sigmoidal profiles with variable lag or induction times are shown by the extinction at 490 nm, relative to the growth of the J-aggregates (Figure 2c). 

**Figure 4:**
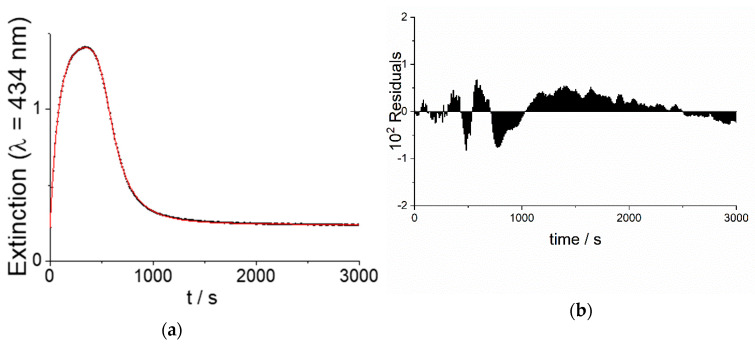
(**a**) Extinction time trace for the demetallation of ZnTPPS_4_ (3 μM) and the consecutive formation of H_2_TPPS_4_ J-aggregates at constant ionic strength (IS = 0.6 M, ZnSO_4_) and [H_2_SO_4_] = 0.05 M. The solid line represents the best-fitted curve to the experimental data according to Equation (3). (**b**) Residuals, accounting for the validity of the model, are shown on the bottom.

These latter data can be analyzed through a model proposed by Pasternack et al. [70,71], that refers to an auto-catalytic growth starting from an initial nucleus or seed with a specific minimum size (*m*, number of porphyrin in the initial seed) and proceeding with a time-dependent catalytic rate constant, *k_c_*, that changes according to a power law of the type *rate* ∝ *t^n^* (*n*, is a characteristic time exponent). The complete model (Equation (2), see Experimental Section) includes an additional rate term, accounting for an uncatalyzed pathway (*k*_0_). The traces at 490 nm display a decrease of the initial lag-time on increasing [H^+^] or [ZnSO_4_], as expected for the aggregation of H_2_TPPS_4_ that is known to be favored at lower pH and higher ionic strength [7]. The extinction traces at 434 nm, relative to the diacid H_2_TPPS_4_, exhibit bell shaped profiles, whose maxima decrease in intensity and move to longer times on decreasing [H^+^] (Figure 2b). The initial growth can be fitted to a mono-exponential function whose rate should reflect that obtained through the corresponding trace at 422 nm. Indeed, apart from the fastest observed rate (i.e., for [H_2_SO_4_] = 0.1 M, [ZnSO_4_] = 0.6 M), the formation of H_2_TPPS_4_ by demetallation of ZnTPPS_4_ and its subsequent conversion to J-aggregate are strongly coupled processes, making the correspondence unreliable. On increasing the concentration of free H_2_TPPS_4_ in solution, self-assembling into J-aggregates takes place and the extinction of the diacid species decreases following sigmoidal profiles, matching the growth of extinction at 490 nm. A modified equation coupling a first order decay and the autocatalytic model (see Experimental Section, Equation (3)) has been developed to fit the extinction at 434 nm. Figure 4 shows an example of the fitted curve to the experimental data, together with the residuals, accounting for the validity of the model. 

**Figure 5:**
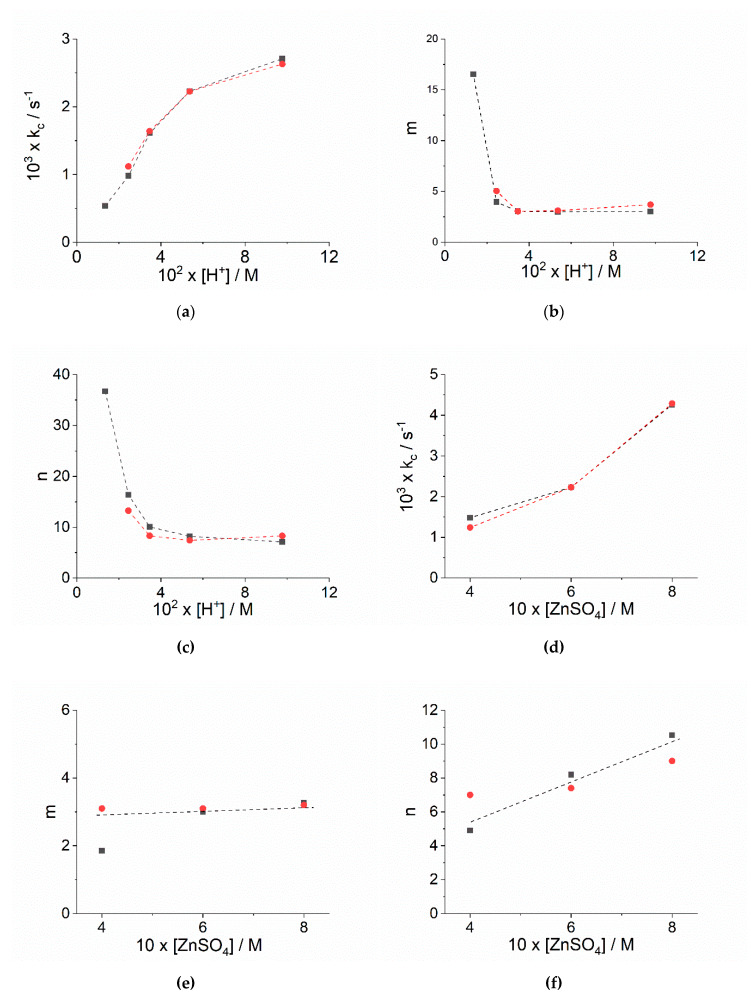
Plot of (**a**,**d**) the rate constants *k_c_* (s^−1^); (**b**,**e**) the size of the critical nucleus, *m*; and (**c**,**f**) the time exponent *n* for the catalyzed growth of J-aggregates as function of [H^+^] at constant ionic strength (upper plots) and as function of [ZnSO_4_] at constant pH ([H_2_SO_4_] = 0.05 M) (lower plots) (black and red data refers to analysis at 490 nm and 434 nm, respectively).

Apart from the slowest kinetic data, where the coupling of the demetallation and aggregation processes prevent a reliable analysis, the obtained parameters are in good agreement with those calculated separately from the data at 422 and 490 nm. The complete kinetic analysis of all traces and the relevant parameters *k_obs1_*, *k*_0_, *k_c_*, *m* and *n*, are listed and compared in Table 1. An evaluation of the latter three is reported in Figure 5a–f. 

An inspection of Figure 5a,d reveals that the rate of the catalytic process *k_c_* increases on increasing both [H^+^] and [ZnSO_4_]. Taking apart the data at lower acid concentration, the size of the critical nucleus, *m*, is on average 3–4, in line with literature data, indicating that the rate-determining step requires the formation of a trimer or tetramer of H_2_TPPS_4_ [56,60]. The values of the time exponent *n* are more largely variable (7–16) with respect to *m*.

In any case, the large deviation observed for the slower kinetics is once again related to the progressive strong interference of the two consecutive processes. The parameters *m* and *n* are deeply related to the nucleation stage and the slower the demetallation rate, the longer the “apparent” lag-time in the 490 nm kinetic traces. This consideration suggests that the corresponding values should be regarded with caution. In all the cases, the uncatalyzed pathway (*k*_0_) makes almost no contribution to the curves.

### 2.2. Chiroptical Properties

**Figure 6:**
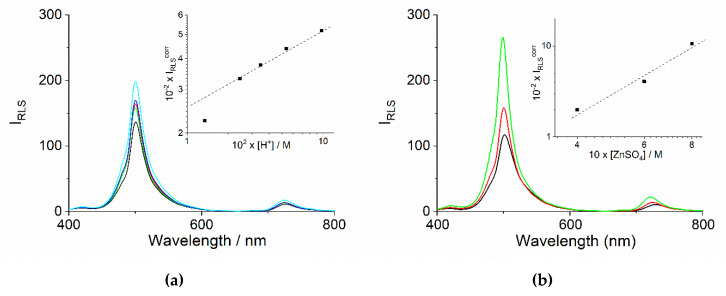
RLS spectra of the samples at the end of the aggregation process for the experiments at variable [H^+^] ([H_2_SO_4_] = 0.01 (black), 0.02 (green), 0.03 (red), 0.05 (blue) and 0.1 M (cyan)) and fixed ionic strength (IS = 0.6 M) (**a**) and at variable ionic strength [ZnSO_4_] = 0.4 M (black), 0.6 M (red) and 0.8 M (green) and fixed [H^+^] ([H_2_SO_4_] = 0.05 M) (**b**). The inset of both frames report the log–log plots of the RLS intensity at maxima corrected for the extinction of the samples as function of [H^+^] (left) and [ZnSO_4_] (right).

Resonance enhanced light scattering occurs when a large number of monomers (N > 25) [72] assemble into aggregates having considerable size, and the chromophoric interacting units are strongly electronically coupled [73]. The formation of J-aggregates in our sample is confirmed by the occurrence of quite intense peaks at about 490 nm in the resonance light scattering (RLS) spectra of the samples at the end of the aggregation process (Figure 6a,b). The magnitude of the RLS effect is related to the scattering and the absorption cross-sections of the aggregate and as a consequence of its size and concentration. The intensity of RLS, corrected for the extinction of the solution, increases on increasing the concentration of both [H^+^] and [ZnSO_4_] (insets of Figure 6a,b). 

**Figure 7:**
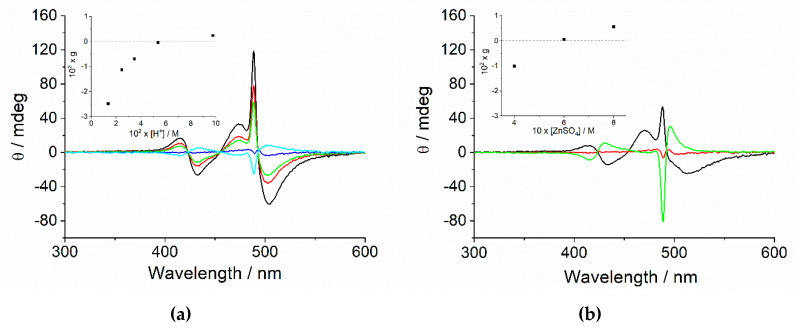
CD spectra of the samples at the end of the aggregation process for the experiments at variable [H^+^] and fixed ionic strength (IS = 0.6 M) ([H_2_SO_4_] = 0.01 (black), 0.02 (red), 0.03 (green), 0.05 (blue) and 0.1 M (cyan)) (**a**) and at variable ionic strength and fixed [H^+^] ([H_2_SO_4_] = 0.05 M) ([ZnSO_4_] = 0.4 (black), 0.6 (red) and 0.8 M (green)). The inset of both frames reports the plots of the asymmetry g-factor at the J-couplet (490 nm) as function of [H^+^] (**a**) and [ZnSO_4_] (**b**).

This finding is in line with an increment of size and number of aggregates in solution, as resulting from the higher stabilization of the nano-assemblies by lowering pH or increasing the ionic strength. At the same time, lower pH values guarantee a faster release of monomeric H_2_TPPS_4_ thus favoring a rapid formation of a larger number of nuclei and consequently of J-aggregates. As already pointed out in previous studies, arrangement of porphyrins in the basic units of these nano-assemblies can be chiral [44,74], even if in the absence of a specific bias, a racemic mixture is expected. Spontaneous symmetry breaking could then lead to an excess of one of the two enantiomorphic aggregates, thus originating detectable circular dichroism bands. Figure 7a,b exhibit the circular dichroism (CD) spectra recorded on the aggregated samples at the end of the kinetic growth. Two distinct groups of signals are present: the most intense is a sharp couplet centered at 490 nm, in correspondence to the J-band, and a less intense couplet centered at 424 nm, corresponding to the usual H-band of these aggregates. Quite intense bands are also evident in the region around 490 nm having a much larger envelope with respect to the peculiar sharp J-couplet. This large feature could be explained in terms of differential scattering arising from the chiral nanoaggregates. As pointed out previously, the extinction spectra of these samples evidence a J-band much broader and it extended over the spectrum, with respect to the normal sharp and intense B-band usually obtained in aggregated samples of H_2_TPPS_4_. 

**Figure 8:**
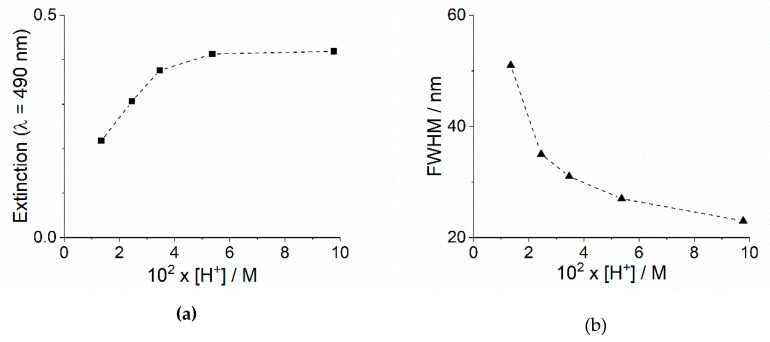
(**a**) Plot of the extinction values and the relative full width at half maximum, (**b**) FWHM for the J-band at 490 nm of the final UV/Vis spectra as function of [H^+^] and fixed ionic strength (IS = 0.6 M).

This experimental evidence has been explained with a dipolar coupling mechanism, more similar to metal colloids, rather than with the Frenkel exciton model, in the case of J-aggregates of the same porphyrin prepared in the presence of a series of cationic polyamines [46,65,75]. Indeed, as aggregation rates increase the J-band becomes more intense and sharp (Figure 8) and correspondingly, the larger band in the CD spectra appears less evident. A similar behavior is displayed on increasing the ionic strength at fixed pH.

**Figure 9:**
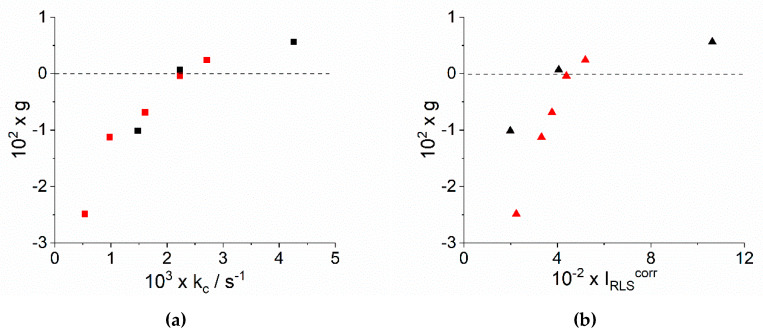
Plot of asymmetry g-factor as function of the corresponding (**a**) rate constants *k_c_* and (**b**) of the RLS intensity at maxima corrected for the extinction of the samples (black points at fixed ionic strength, red points at fixed pH).

Most importantly, all the spectra at high pH or at lower ionic strength display a negative Cotton effect on the entire band. On increasing [H^+^] or [ZnSO_4_] the absolute intensity of the bands decreases and the bands eventually show a positive Cotton effect. This behavior is rather peculiar, since in J-aggregates obtained by fostering aggregation through simple acid addition (i.e., HCl or H_2_SO_4_, no salt added), positive Cotton effects are statistically predominant [76]. Furthermore, an acceleration in the aggregation process has been related to a complete loss of optical activity in solution. [56] In the present case, the asymmetry g-factor (g = Δε/ε) when plotted against *k_c_* clearly evidences a decrease in the absolute value, nulling and actually inverting sign for higher rate values (Figure 9a). The inversion of CD sign is not unprecedented, since in our previous investigations on the role of adventitious trace of zinc(II) metal ions during the self-assembly formation, we proved that addition of a tiny amount of ZnTPPS_4_ (about 5%) is able to drive the enantiomeric excess from a positive to a negative Cotton effect [60]. Another intriguing difference to literature data is given by the dependence of g-factor from the intensity of RLS (Figure 9b).

**Figure 10:**
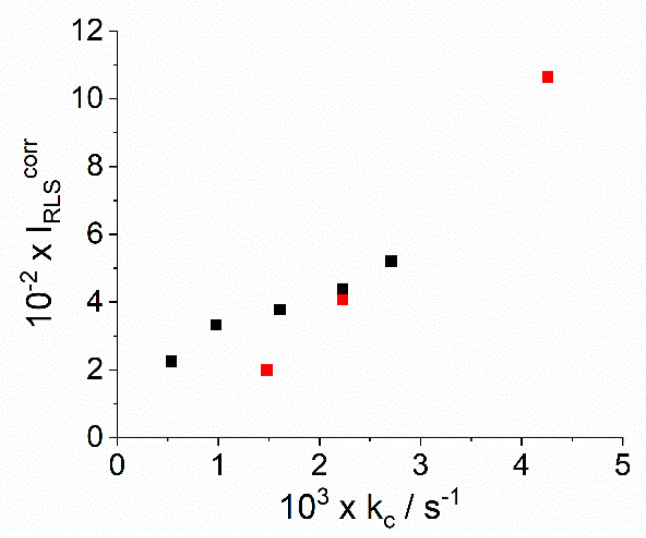
Plot of the RLS intensity at maxima corrected for the extinction of the samples as function of the corresponding rate constants *k_c_* (black points at fixed ionic strength, red points at fixed pH).

In the case of J-aggregates prepared by HCl addition, we reported that g-factor decreases with the intensity of RLS, obeying a scaling law of the type *g* ∝ *I_RLS_^β^* [56]. Here, samples with smaller scattering intensity exhibit larger negative CD effect, that decreases progressively on increasing *I_RLS_* till nulling and eventually inverting the sign of g. All the experimental evidence suggests that zinc cations are playing a specific role not merely controlling the ionic strength of the medium. They have an impact on: (i) the extinction spectra contributing to changing the electronic coupling mechanism determining the final appearance of the J-band, (ii) on the enveloping of the CD signals and (iii) on the sign and magnitude of the Cotton effect. Contrary to anionic species that exert a role on the kinetics of this supramolecular assembly formation [76], a cation is expected to interact strongly with a negatively charged porphyrin. Even if, after protonation the diacid porphyrin could be considered as a neutral zwitterion, some pre-interaction with the metal ions could still exert an impact on the early stage of the process. As both nucleation and growth dynamics are crucial in the aggregation of colloidal systems, the presence of zinc ions could drive the kinetics to different final supramolecular structures [4]. Actually, the initial nucleus size (*m* = 3–4) is very close to that reported for the formation of nanotubes driven only by inorganic acids. So the metal cations could play a role in the mesoscopic arrangement of the system, analogously to cationic polyamines, that lead to very broad J-bands [39,40]. To confirm a potential structural role of zinc cations on the network, we observe that the intensity of RLS increases with increasing the rate for the catalyzed pathway (Figure 10). This behavior is opposite to that reported for aggregation in the presence of simple inorganic acids at low pH, suggesting an involvement of the metal ions in structuring J-aggregates and not only on the rate determining step or nucleation [56,74].

## 3. Materials and Methods

### 3.1. Materials

5,10,15,20-tetrakis(4-sulfonatophenyl)porphyrin (TPPS_4_) sodium salt was obtained from Aldrich. Zinc(II) sulfate heptahydrate, zinc(II) oxide (Aldrich, Milan, Italy) and sulfuric acid (Fluka, Milan, Italy) were of the highest commercial grade available and were used as received without further purification. Zn(II) metal derivative of TPPS_4_ (ZnTPPS_4_) was prepared at room temperature using a heterogeneous metallation procedure with ZnO. In particular, an aqueous solution of TPPS_4_ (≈5 mM) was refluxed in a two necked flask in the presence of a small amount of solid ZnO. Small aliquots of this solution were sampled every 5 min. After properly diluting with water in a UV/Vis cell, the eventual conversion was spectrophotometrically monitored by the peculiar spectral changes in the Q-band region. Zinc(II) insertion into the porphyrin core causes a change from the usual four-band pattern (516, 554, 580, and 634 nm, D_2h_ symmetry) to a two-band pattern (556 nm and 598 nm, D_4h_ symmetry). After reaction was complete, the solution was cooled down to room temperature and the residual amount of ZnO was removed by filtering the solution through a Millipore 0.22 μm syringe filter. The purity of the samples were further assessed bye fluorescence emission spectroscopy by checking for the absence of any residual emission from the parent metal-free porphyrin (644 nm and 708 nm, this latter being more diagnostic).

All the solutions were prepared in high-purity doubly distilled water from Galenica Senese (Siena, Italy). The porphyrin concentration used in our experiments was determined spectrophotometrically using the molar extinction coefficients at the Soret maxima (TPPS_4_: 5.33 × 10^5^ M^−1^ cm^−1^, λ = 412 nm; ZnTPPS_4_: 6.83 × 10^5^ M^−1^ cm^−1^, λ = 422 nm). 

### 3.2. Methods

UV/vis extinction spectra were obtained on an Agilent 8453 diode array spectrophotometer. A UV filter (Hoya glass type UV-34, cut-off: 340 nm, Milan, Italy) was placed in front of the measurement cell to prevent photo-degradation of porphyrins for prolonged exposition during the kinetic runs. Resonance light scattering (RLS) spectra were acquired on a Jasco FP-750 spectrofluorimeter, using a synchronous scan protocol and a right angle geometry [73]. Circular dichroism (CD) spectra were carried out on a Jasco J-710 spectropolarimeter (Milan, Italy). An estimate of the CD oscillator quality is accomplished by the dissymmetry factor g = Δε/ε = ΔA/A, where ΔA = θ/32,980 (ΔA is in absorbance units and θ is the ellipticity in mdeg).

Kinetic experiments were performed by collecting spectra from the reacting solutions placed in the thermostatic sample holder of the spectrophotometer. The temperature was kept fixed at 298 K during the kinetic runs through an external recirculating water bath. All the reactions were started by adding a known aliquot of acid solution to a prediluted ZnTPPS_4_ solution (3 μM) in the presence of ZnSO_4_ at the required ionic strength and the mixture was inverted three times to ensure mixing of reactants. A large excess (>100-fold) of sulfuric acid over porphyrin ensured pseudo-first order conditions in any kinetic run. In order to obtain the kinetic parameters, extinction data vs. time were extracted from the collected spectra at selected wavelengths. The demetallation reactions abided by a first-order law and the observed rate constant (*k_obs1_*, s^−1^) was obtained either from a non-linear least square fit of the experimental extinction data at 422 nm to the Equation (1a,b). The aggregation process follows a sigmoidal profile that can be treated by an autocatalytic model proposed by Pasternack et al. [70,71]. The experimental extinction data at 490 nm were analyzed by a non-linear least square fit to the Equation (2). The two consecutive processes, in other words, demetallation and aggregation, are generally coupled and an analysis of the extinction data at 434 nm is difficult especially if demetallation is slow and the diacid porphyrin is still released by ZnTPPS_4_, when aggregation is already in progress. In order to confirm the kinetic parameters obtained by the separate analysis of the data at 422 and 490 nm, we performed a non-linear least square fit of the extinction data at 434 nm to the Equation (3). Considering the large number of parameters,some of those were initially fixed using the values obtained from the separate fitting procedures. In most of the kinetic analyses *k*_0_ made an almost negligible contribution to the curve, and the parameter *t*_0_ was close to zero.

Ext_t_ = Ext_0_ + (Ext_0_ − Ext_∞_) × exp(−*k_obs1_* × *t*) (1a)

ln (Ext_t_ − Ext_∞_)/(Ext_0_ − Ext_∞_) vs*. t* (1b)

where Ext_0_, Ext_∞_ and *k_obs1_* are the parameters to be optimized (Ext_t_ = the extinction at time t; Ext_0_ = extinction soon after mixing reagents; Ext_∞_ = extinction at completion of the reaction), or from the slope of the semi-logarithm plot of Equation (1b).

Ext_t_ = Ext_∞_ + (Ext_0_ − Ext_∞_) (1 + (*m* − 1) {*k*_0_t + (*n* + 1)^−1^ (*k_c_* t)*^n^*^+ 1^})^−1/(*m*−1)^ (2)

where Ext_0_, Ext_∞_, *k*_0_, *k_c_*, *m* and *n* are the parameters to be optimized.

Ext_t_ = Ext_∞_ + *A* exp (−*k_obs1_* × *t*) + (Ext_0_ − Ext_∞_) (1 + (*m* − 1){*k*_0_(*t* + *t*_0_) + (*n* + 1)^−1^ (*k_c_* (*t* + *t*_0_))*^n^*^+ 1^})^−1/(*m*−1)^ (3)

where Ext_0_, Ext_∞_, *A*, t_0_, *k_obs1_*, *k*_0_, *k_c_*, *m* and *n* are the parameters to be optimized (Ext_0_ = extinction soon after mixing reagents; Ext_∞_ = extinction at completion of the reaction; *A* is the extinction difference at the end of the demetallation kinetic and at *t* = 0; *t*_0_ is an offset parameter taking into account the onset of aggregation).

## 4. Conclusions

The zinc derivative of TPPS_4_ offers an easy way to precisely control the supramolecular assembling pathway to J-aggregates, affording a quite reproducible monomeric initial state. A proper choice of pH and ionic strength in solution allows varying the concentration of the initial building block, in other words, the diacid H_2_TPPS_4_. When a zinc(II) salt is added to foster aggregation under acidic conditions, J-aggregates with distinctive electronic and chiroptical properties are obtained. The exact role of zinc cations still remains unclear, but this species is involved in the stabilization of the nanoaggregates and in the way they adapt to building a mesoscopic network, responsible for the peculiar dipolar coupling in electronic extinction and CD spectra. At difference with J-aggregates obtained through inorganic acids, here the sign of the Cotton effect induced in the absorption bands can be switched from negative to positive by changing pH and salt concentration. All together, these findings give further insights into the properties of these intriguing supramolecular assemblies and open the way to achieve new ways to control and tune their spectroscopic properties for potential applications.

## Figures and Tables

**Table 1:** Relevant kinetic parameters for the demetallation of ZnTPPS_4_ (*k_obs1_*) and aggregation of H_2_TPPS_4_ in J-aggregates (*k_c_*, *m* and *n*) as function of [H^+^] and ionic strength (T = 298 K).

10^3^ [H^+^]/M	10^3^ k_obs1_/s^−1 a^	10^3^ k_c_/s^−1 b^	m^b^	n^b^	10^3^ k_c_/s^−1 c^	m_c_	m_c_
13.5 ^d^	0.261 ± 0.001	0.537 ± 0.002	16.5 ± 2.7	36.7 ± 6.3	^e^	^e^	^e^
24.6 ^d^	1.29 ± 0.01	0.979 ± 0.001	3.9 ± 0.1	16.4 ± 0.4	1.12 ± 0.38	5.0 ± 0.9	13.3 ± 5.8
34.7 ^d^	3.55 ± 0.02	1.61 ± 0.01	3.1 ± 0.1	10.0 ± 0.1	1.64 ± 0.60	3.0 ± 0.3	8.3 ± 3.6
53.7 ^d^	9.76 ± 0.05	2.23 ± 0.01	3.0 ± 0.1	8.2 ± 0.1	2.23 ± 0.02	3.1 ± 0.1	7.4 ± 0.2
97.7 ^d^	54.7 ± 0.7	2.71 ± 0.01	3.0 ± 0.1	7.1 ± 0.1	2.63 ± 0.20	3.7 ± 0.1	8.3 ± 0.9
53.7 ^f^	12.3 ± 0.1	1.48 ± 0.01	1.8 ± 0.1	4.9 ± 0.1	1.24 ± 0.26	3.1 ± 0.3	7.0 ± 1.9
53.7 ^g^	9.59 ± 0.08	2.23 ± 0.01	3.0 ± 0.1	8.2 ± 0.2	2.23 ± 0.60	3.1 ± 0.3	7.4 ± 2.6
53.7 ^h^	10.8 ± 0.1	4.26 ± 0.01	3.3 ± 0.1	10.5 ± 0.1	4.29 ± 0.99	3.2 ± 0.2	9.0 ± 2.4

^a^ from linear fit of extinction data at 422 nm (see Experimental Section). ^b^ from non-linear fit of extinction data to Equation (2) at 490 nm. ^c^ from non-linear fit of extinction data to Equation (3) at 434 nm. ^d^ IS = 0.6M [ZnSO_4_]. ^e^ not reported due to unreliable fit. ^f^ [ZnSO_4_] = 0.4 M. ^g^ [ZnSO_4_] = 0.6 M. ^h^ [ZnSO_4_] = 0.8 M.
